# Mental Evolution and the Insanities of To-Day

**Published:** 1910-10-29

**Authors:** Robert Jones


### Hospital Clinics.

### MENTAL EVOLUTION AND THE INSANITIES OF TO-DAY.

By ROBERT JONES, M.D., F.R.C.P., F.R.C.S.

(A Lecture delivered at the West London Post-Graduates' College, Hammersmith, on June 17, 1910.)

Gentlemen,—The subject of my lecture—or perhaps I may call it a demonstration—is a study of the varieties of insanity as they are known to-day. But, in the first place, I desire to say a few words about the anatomy of the brain. I have here the right hemisphere of the brain of a lunatic. The first thing we observe is the fissure of Sylvius, fairly oblique, and in this case not quite horizontal in direction, also showing the development of the parietal and the frontal lobes. This fissure of Sylvius begins to develop in the fourth month of intra-uterine life. The next feature that claims our attention is the sulcus of Rolando, in a mid-position between the ascending frontal and the ascending parietal. Here we have the motor part for the leg and the arm, and the sensory part opposite to it; also the motor parts for the face, tongue, and lips, and the parts corresponding to them sensorially. The next most important sense, and the one which gives most information, is that of the hearing. It is much more informing than the sense of sight. We should not be able to speak or to appreciate the niceties of language were it not for the sense of hearing. That is why a child contracting deafness after scarlet fever or some such condition in early life may become a deaf-mute. Here we have the most highly evolutionised and the most complex sense, and, therefore, the one which it is easiest to disorganise. Insanity attacks the least organised and the most highly evolutionised centres. For that reason hallucinations connected with the sense of hearing are very common in insane people. The hearing centre is situated, roughly, between the two ends of the fissure of Sylvius. In these transverse gyri we have the "arrival platform" of hearing sensations, and all around this part the messages are elaborated by the pyramidal cells. Inflammation and adhesions of the meninges round this area will certainly affect the sense of hearing. Turning to the median aspect of the brain we come upon the area of the sense of sight. This is situated in and around the calcarine fissure. In monkeys and the lower animals the calcarine sulcus is more extensive, and appears on the outer surface of the brain, but in man this part rarely extends beyond the occipital pole outwardly. Then we have on the cortex of the brain that most important angular gyrus connecting the visual with the hearing centres.

### Some Anatomical Points.

The blood-vessels go directly into this cortex and end in capillaries and veins, and practically if we have blocking of the blood-vessels in a cortical area we throw out of circuit the whole of that convolution. The next most important thing to be noted is the lymphatic space. The lymphatic system of the brain is very important; changes are constantly going on of a bio-chemical nature in the pyramidal cells, and the products of their decomposition are carried into the lymphatic channels by the glia, or spider, cells. Above the pyramidal cortical cells we have a fibre layer just under the pia mater, which is called the "tangential" layer, and this is a layer of association neurons. Below the pyramidal cells is a layer of granular neurons which constitute the "arrival platform" of the different sensations.

So much for the anatomy. It is important now to consider the brain of man from two standpoints—in the first place, phylo-genetically; and in the second, onto-genetically. This consideration come in because it is the tendency for the offspring to inherit the organic structure of the parent or the race. Man is a vertebrate animal, with animal instincts and ancestral traditions. I regard the instincts as those conditions which are fundamental and do not require educating. Such are the stimulus of sex, the craving for food, the search for warmth. Now, the brain, when it dissolves, dissolves in the reverse order of its evolution, the association neurons being the first to go. The inhibition is thus destroyed, the judgment is affected, and, finally, with the dissolution of the last layer, the sensation itself is abolished and the instincts reversed or perverted.

The influence of environment is an important factor in insanity. There are some who tell us that heredity is the principal factor and environment merely secondary, but I have come to the conclusion that environment has a great deal to do with the mental states. I do not say that the influences of environment are immediately acquired, but they are acquired in the course of time. We have examples of this influence of environment in the vegetable kingdom. Alpine plants, for example, transplanted to the plains assume plain characters and *are true to the seeds*. The environment in the long run determines the direction of the plant's evolution. It may be said, therefore, that every person is sane or insane in relation to his own standard and his own environment. The environment must always be considered in estimating the insanity. Indeed, insanity has been defined as inability to react normally to changing conditions of environment.

### The Mechanism of Mind.

What is the mechanism of mind? What happens when we think? We depend upon our experience for our knowledge, and experience comes through the avenues of the senses. An object is presented to the sight or the hearing and is perceived, and, moreover, that perception can be brought back even when the object itself has been removed. The mind can react both to objects as perceptions and to the memories of objects as ideas. We may make mistakes in appreciating or perceiving a thing, and this erroneous perception is called an illusion. But when the thing has been removed from the presence of the particular sense-organ involved, the erroneous impression may be revived in the memory and presented again as a hallucination. We know well that these disorders of perception and ideas are important because they affect conduct, but it is not the illusions or hallucinations or delusions as such which constitute insanity. Many people suffer from illusions, but we should never dream of calling them insane. A person who sees mocking faces in the moon is suffering from an illusion; Pepper's Ghost—much talked about in my boyhood—and the Spirit of the Brocken are illusions; the sensation of rocking ground which some people experience immediately after a sea voyage, or the sensation of motion when a train passes the observer while he is seated in another train which is standing still, these are all illusions, but no one would state that people who are suffering from these illusions are necessarily insane.

We have a classic example of visual illusions in Holy Writ (Daniel v. 25), after the excessive drinking at the feast of Belshazzar, when the words "Mene, Mene, Tekel, Upharsin" were seen written on the wall of the great banqueting hall. Dr. Johnson suffered from hallucinations. He used to hear his mother's voice from Lichfield calling out "Sam" when he was at Pembroke College, Oxford. But hallucinations, although often quite harmless, are dangerous symptoms nevertheless, because they may influence conduct, and aural hallucinations may be so dominating and so tyrannical as not infrequently to give rise to acts of extreme and dangerous violence. The poet Cowper was distracted by illusions, and wrote "John Gilpin" during an attack of intense melancholia. The great Esquirol saw "monks like cats capering about the monastery," while Napoleon used to have interviews with a familiar spirit in the shape of "a little red man." Joan of Arc, Peter the Great, and Warren Hastings are cited as names of persons of no common mental powers who nevertheless suffered from delusions, although they were not regarded as insane.

### Factors in Causation.

Insanity is the product of two factors—stress and heredity. It is no new disease. It was known before the Christian era. We have it recorded (1 Sam. xxi. 13 and 14) that King David feigned madness in Gath. "He scrabbled on the doors of the gates and let his spittle fall down upon his beard." Races differ very much among themselves in their respective liability to insanity. It has been suggested that the Jews are more prone to insanity than are other races, and that the Teutons are less subject than some of the Latin nations. The vivid imagination of the Celt, as we know, often carries him astray.

In considering any classification of the insanities it would be very desirable to construct a scheme having a psychological basis. But it cannot be done. We cannot have a psychological classification because, for one reason, illusions may exist without an affection of the judgment or will-power of the person concerned. In a case of acute mania we have, on the other hand, hallucinations in abundance, and the most assertive illusions, with other powers of the mind quite distorted and the will-power also in abeyance. Next in order of desirability would be a pathological classification, in the same fashion that we classify morbid conditions of the respiratory system, inflammation of the lungs, pleurisy, and so on. But such a scheme is not complete. The researches of chemistry and microscopical investigations have not yet availed us adequately for this purpose. The most serviceable and the best classification for us at present is the "symptomatological" one. We often cannot do more than treat symptoms.

### Varieties of Insanity.

Taking, then, classification based upon symptoms, we have, in the first place, the condition of mania, in which the patient is boisterous and violent; melancholia, in which he is depressed; dementia, which is the goal of many cases of both mania and melancholia; and that ever-fascinating variety of insanity, looked upon as a mental stigma, and called paranoia, or chronic delusional insanity, meaning simply systematised delusions. There are multitudes of people about who are suffering from insanity in this last-named form. I met one of them casually in Westminster Abbey not very long ago. He told me that I was in league with his persecutors, and that now he had me. This perversion of the normal mind is met with most frequently in those who may be described as eccentrics, oddities, cranks, mono-maniacs, people who will pass a resolution even against the law of gravitation! But it is a very interesting condition. Moreover, it has an atavistic element. When man was single handed and had to combat the forces of Nature, the sun and storm and sea and rain were all personified. And these patients "hark back" to this condition of things. They invent new and mysterious names to personify and explain the influence of unseen agencies upon their bodies. Next we have the teratological condition, which is represented by imbecility and idiocy. These are spontaneous variations, and nobody knows how they can be accounted for. We may have two mentally healthy parents and they beget an idiot child. Among imbeciles there is a class which has been discovered by the Royal Commission on the Feeble-minded—namely, that of the moral imbecile. The variety was known to experts long before, of course, but it was discovered for the public by that Commission. The various Education Committees have medical men who seek out mentally deficient children, and they find no small number of these moral imbeciles. They are plausible little children, often very clever, but they will "lie like truth," and they will indulge in such acts as throwing the cat on the fire, or pinch other children, or destroy their mother's jewellery! This class is quite well known to specialists.

### Dementia Precox.

Of all the forms of mental disorder of the present day the most important is insanity in young people—the condition which Esquirol has described as "acquired imbecility," and by later writers as dementia precox, or adolescent insanity. I know several young men who have distinguished themselves at competitive examinations who have fallen victims to this form of insanity. It was certainly known a hundred years ago, and described in 1798 by Haslam, physician to Bethlem, who referred to it as "a species of insanity sometimes occurring about the time of puberty, especially in those who have possessed a good capacity, a lively disposition, and in females more than in males. Their faculties are gradually obliterated until they are at last complete and incurable idiots." This form of insanity attacks prematurely our most promising youths, generally the precocious ones, and the brain worker rather than the manual worker. The physical explanation has been offered that we can only go as far as the inherent durability of our neurons will allow us. The failure of that durability may cause a breakdown at the age of fifteen, or, if the mechanism is better, at the age of twenty, or, if better still, at the age of thirty, and so on. If there is marked hereditary influence predisposing to failure in the biochemical nature of the pyramidal cells, a very little stress will be sufficient to occasion a mental breakdown. But I cannot recall a single instance of a patient coming to Claybury having broken down purely from overwork alone. There is always some other associated factor. I think that hard work on the whole is a good thing. The brain is most active when the greatest number of stimuli are coming into it from the outside world. Of course, I must qualify this by saying that a man must not burn the candle at both ends. Over-exercise, over-strain, over-fatigue, and over-excitement are responsible for a good deal of nervous disease, but nevertheless the man whose working days are ordered by regular habits, even if the amount of work is very considerable, hardly ever or very rarely breaks down. The Lunacy Commissioners demonstrate by figures in their most recent report that the variety of mental disease which went by the name of acquired imbecility is now more common than formerly. These cases almost invariably commence with some depression. The disease has presumably a chemical pathology, and very careful researches made in the laboratory at Claybury by Koch and Mann have seemed to prove the theory that it may be referred to some chemical metabolism. Microscopically the pyramidal cells show a deficiency both in number and in the amount of chromophilous substance, and there is an increase, in the terminal stages, of glia tissue. The proportion of these cases has risen from 21.5 per cent. to 31.2 per cent. of all cases of insanity during twenty-five years.

### The Senile Variety.

The next insanity to which I would direct your attention is the senile variety. It is a great thing to be able to grow old physiologically. Senile insanity is really closely connected with arteriosclerosis. One patient, for example, was sent to us recently as an epileptic, but he had had no fits before the age of twenty, which, speaking generally, ruled out epilepsy, and an examination of his arteries solved the diagnosis—he had commencing arterio-sclerosis. The frequency of the pre-senile cases suggests the truth of the "too old at forty" aphorism. Last year 1,156 of these cases were received into the asylums of England and Wales, and senile insanity has increased from 11 per cent. to 20 per cent. in our asylums during a period of fifteen years. It is possible that the Old-Age Pension Act may ultimately be a means of lowering the number of admissions, owing to a desire to keep weak-minded old people at home.

The next variety includes cases of insanity which commence in neurasthenia and psychasthenia. The Report of last year already referred to states that in more than 200 cases these causes were given. Mental stress is reported to have caused insanity in 4,410 persons, these cases being an indication of excesses of many kinds. They are due to the struggle for existence, and also probably to the marriage of the unfit. The remedy is partly to be found in a more temperate life, and partly also in the compulsory notification of syphilis—a matter about which those of you who are engaged in the Services know far more than myself. Last year, in the case of 850 males and 153 females, syphilis often indicated by night headaches was stated to be a factor in the production of insanity. But "civilisation" as "progress" is responsible also for the manufacture of lunatics. An individual, let us say, tries for a certain prize, but another competitor obtains it. Disappointment and shame then react upon him, and the same circumstances are repeated time after time until he gradually sinks to the level of the unsuccessful and the unemployed. Each year the standard of efficiency becomes higher, and those who cannot keep pace with the demands of our twentieth century, assist in many cases to swell our lunatic population.

### G. P. I.

General paralysis of the insane, which is associated very closely with syphilis, was responsible for the admission to asylums of 1,512 persons. Something can be done for tabes, to which these cases are closely related. We know that in tabes we get posterior horn fibres affected, and we can do a great deal of good by educating them. For example, one ordinarily walks automatically, but the man suffering from locomotor ataxia has difficulty in walking. In such a case we can take advantage of the pyramidal cells and give the patient the "idea" of walking with his head. He has previously walked automatically with his spine; he now begins to walk with his brain, and this treatment is very successful. This paralytic insanity is closely connected with syphilis. Persons who indulge in alcohol to excess, and who have also contracted syphilis, are more liable to general paralysis following upon injuries to the head. I gave evidence not long ago in the case of a claim for compensation made on behalf of the friends of a man who had died of general paralysis of the insane after a slight accident to the head. It was found that he had a history of gradual failure for two years, and a letter was presented from the father to the effect that his son ought to have been where he was (at Claybury) eighteen months before. He had certainly had symptoms for nine months previously. Two months before admission to the asylum he suffered a slight injury to the head, was attended at the hospital, and was at work again on the following day. I am unwilling to put down officially as a definitely ascertained factor of causation the occurrence of a head injury, because not infrequently the patient has temporarily recovered and worked after the accident, and such a record would probably multiply cases of appeal for compensation, but a note of the occurrence is made in our case-books.

### Alcohol and Insanity.

It is rather remarkable that after all the preaching about temperance the cases of insanity associated with alcohol show an increase. According to the last Report, 2,446 men and 992 women of this class were admitted into the asylums of England and Wales whose insanity was associated with drink. Epilepsy was seen in 7 per cent. of the male admissions, and in 5 per cent. of the female. It is interesting to note that cases of insanity after surgical operations appear to be on the increase. The number of admissions last year from this cause is given as 106. A very important association of insanity is heart disease and arterio-sclerosis, and I cannot help thinking that we now find arterio-sclerosis much more commonly than we did years ago. Cardio-vascular conditions and renal disease were responsible as a factor of insanity in 837 cases, and 459 persons died in asylums from renal disease within the same year.

### Influenza and Other Causes.

The influenza bacillus is a nerve toxin next in importance, I believe, to the treponema pallidum as a specific cause of insanity. Influenza, which has been described as responsible for lowering the nerve power of one-third of Western Europe, is reported as an associated cause of insanity in 568 cases. Tuberculosis figures as a cause in 183, but 1,332 persons died from this disease in asylums during the same year. There is, indeed, a very high mortality from tuberculosis in asylums. It has been stated to be ten times as high within these institutions as it is among the population outside, and this in spite of the fact that we, asylum physicians, do all we can to keep the people out of doors and under healthy conditions. But the demented person often sits or stands in the same position, he is chilled in winter and he is rarely warm in summer, he only takes exercise when led about or assisted, his vitality is low, his respirations are shallow, there is never full entry of air into his lungs, and he is thus predisposed to tuberculosis, but the attendants and nurses in our asylums do not show any particular tendency to contract the disease.

As to the other associated conditions, goître is occasionally found with insanity, also migraine and headaches and skin diseases. One girl who had lived abroad was admitted to the asylum suffering from leprosy, which greatly disfigured her. Her appearance preyed upon her mind, and she was admitted into Claybury. I am glad that the question of sleep in young people has engaged more general attention of late. Our head masters and mistresses are certainly alive to the necessity for proper rest for growing boys and girls, and the hours allotted for sleep in the preparatory and the public schools are an important factor in mental health.

